# Preclinical Assessment of Nebulized Surfactant Delivered through Neonatal High Flow Nasal Cannula Respiratory Support

**DOI:** 10.3390/pharmaceutics14051093

**Published:** 2022-05-20

**Authors:** Francesca Ricci, Arianna Mersanne, Matteo Storti, Marcello Nutini, Giulia Pellicelli, Angelo Carini, Ilaria Milesi, Marta Lombardini, Raffaele L. Dellacà, Merran A. Thomson, Xabier Murgia, Anna Lavizzari, Federico Bianco, Fabrizio Salomone

**Affiliations:** 1 Department of Preclinical Pharmacology, R&D, Chiesi Farmaceutici S.p.A., 15739 Parma, Italy; f.ricci@chiesi.com (F.R.); a.mersanne.stage@chiesi.com (A.M.); m.storti@chiesi.com (M.S.); m.nutini@chiesi.com (M.N.); g.pellicelli@chiesi.com (G.P.); a.carini@chiesi.com (A.C.); i.milesi@chiesi.com (I.M.); m.lombardini@chiesi.com (M.L.); f.bianco@chiesi.com (F.B.); 2 TechRes Lab, Dipartimento di Elettronica, Informazione e Bioingegneria (DEIB), Politecnico di Milano University, 20133 Milan, Italy; raffaele.dellaca@polimi.it; 3 Hillingdon Hospitals NHS Foundation Trust, Uxbridge UB8 3NN, UK; merranthomson@zen.co.uk; 4 Scientific Consultant, 48640 Bilbao, Spain; xabi_murgia@hotmail.com; 5 Fondazione IRCCS Cà Granda Ospedale Maggiore Policlinico, 20122 Milan, Italy; anna.lavizzari@gmail.com

**Keywords:** *Poractant alfa*, eFlow Neos, respiratory distress syndrome, high flow nasal cannula, non-invasive ventilation, nebulized surfactant, aerosol delivery

## Abstract

High-flow nasal cannula (HFNC) is a non-invasive respiratory support (NRS) modality to treat premature infants with respiratory distress syndrome (RDS). The delivery of nebulized surfactant during NRS would represent a truly non-invasive method of surfactant administration and could reduce NRS failure rates. However, the delivery efficiency of nebulized surfactant during HFNC has not been evaluated in vitro or in animal models of respiratory distress. We, therefore, performed first a benchmark study to compare the surfactant lung dose delivered by commercially available neonatal nasal cannulas (NCs) and HFNC circuits commonly used in neonatal intensive care units. Then, the pulmonary effect of nebulized surfactant delivered via HFNC was investigated in spontaneously breathing rabbits with induced respiratory distress. The benchmark study revealed the surfactant lung dose to be relatively low for both types of NCs tested (Westmed NCs 0.5 ± 0.45%; Fisher & Paykel NCs 1.8 ± 1.9% of a nominal dose of 200 mg/kg of *Poractant alfa*). The modest lung doses achieved in the benchmark study are compatible with the lack of the effect of nebulized surfactant in vivo (400 mg/kg), where arterial oxygenation and lung mechanics did not improve and were significantly worse than the intratracheal instillation of surfactant. The results from the present study indicate a relatively low lung surfactant dose and negligible effect on pulmonary function in terms of arterial oxygenation and lung mechanics. This negligible effect can, for the greater part, be explained by the high impaction of aerosol particles in the ventilation circuit and upper airways due to the high air flows used during HFNC.

## 1. Introduction

Non-invasive respiratory support (NRS) is the primary treatment option for spontaneously breathing preterm infants at high risk of developing neonatal respiratory distress syndrome (RDS) [1,2]. Among the available NRS types, the use of the humidified high-flow nasal cannula (HFNC) has gained popularity across neonatal intensive care units (NICUs) due to its ease of use, patient comfort, reduced nasal trauma, and better parent–infant bonding [3,4,5]. HFNC delivers heated, humidified gases through nasal cannulas (NCs) at flow rates ranging between 3 and 8 L/min [6]. Optimal gas humidification, the flushing of the nasopharyngeal dead space, the reduced work of breathing, and the delivery of a distending pressure have been endorsed as the main advantages of this NRS modality [4,5]. Contrary to pressure-controlled NRS devices, such as nasal continuous positive airway pressure (CPAP), the pressure delivered during HFNC is not continuously measured, although it correlates with the preset air flow rate [6,7].

The use of NRS reduces the need for mechanical ventilation and intratracheal surfactant administration. Unfortunately, a significant number of spontaneously breathing preterm infants fail NRS and require mechanical ventilation and/or late intratracheal surfactant. For instance, CPAP failure has been reported to occur in over 40% of infants with a gestational age (GA) below 29 weeks [8,9]. Similarly, Lee et al. reported a 30% HFNC failure in a cohort of infants (n = 97) with a GA > 30 weeks, who eventually required other types of NRS or mechanical ventilation [10]. These preterm infants usually receive intratracheal surfactant later in the course of RDS, when the benefits of therapy may be attenuated compared with the early surfactant administration [11].

The aerosol delivery of surfactant during NRS would represent a non-invasive method of surfactant administration, enabling timely surfactant treatment [12]. Studies in animal models of induced respiratory distress supported with CPAP have demonstrated that nebulized surfactant can be as effective as surfactant instillation in improving arterial gas exchange and lung mechanics [13,14,15]. Moreover, scintigraphy studies confirmed the feasibility of surfactant nebulization during CPAP, revealing a mean lung deposition of 11.4% in healthy non-human primates [16] and 15.9% in newborn piglets [17]. 

Early pilot clinical studies on surfactant nebulization during CPAP demonstrated the feasibility and the safety of this therapy [18,19,20]. However, these initial studies were rather heterogeneous, used jet nebulizers, different surfactant preparations and administration protocols, and included a small number of patients with various GAs. Nevertheless, the lack of a consistent pulmonary response across studies evidenced the poor aerosol delivery efficiency in neonates managed with NRS. Since then, the emergence of the vibrating-membrane nebulizer technology and increased awareness of the intrinsic (e.g., low lung volumes and small airways) and the extrinsic (e.g., NRS modality and bias flow, nebulizer type, and position) factors that influence aerosol lung deposition in neonates [12,21] has led to the development of customized aerosol delivery strategies for this patient population [13,22,23,24]. In this regard, recently published clinical trials on surfactant nebulization have reported encouraging results [25,26]. Unfortunately, the clinical benefits of nebulized surfactant (i.e., the reduction in intubation) were only observed for the most mature neonates (GA > 31 weeks). While CPAP has been almost exclusively used in all clinical studies, a recent single-center, Phase II trial on nebulized surfactant has reported the use of HFNC in a subgroup of neonates [27]. Nevertheless, to the best of our knowledge, neither the lung surfactant dose nor the effect of nebulized surfactant delivered via HFNC has been yet addressed in benchmark studies or animal models of respiratory distress. 

Therefore, the present study was designed to investigate the lung dose and the in vivo efficacy of nebulized surfactant delivered with the investigational eFlow Neos vibrating-membrane nebulizer during HFNC. First, we tested the compatibility of different commercially available NCs commonly used in the neonatal intensive care units (NICUs) with our in vivo setting (spontaneously breathing adult rabbits). After that, we conducted a benchmark study with compatible NCs to compare the nebulized surfactant lung dose under simulated neonatal HFNC. Finally, the effect of nebulized surfactant delivered via HFNC was investigated in spontaneously breathing rabbits with induced respiratory distress.

## 2. Materials and Methods 

### 2.1. Surfactant Preparation and Nebulizer

The natural surfactant *Poractant alfa* was used in the present study (Curosurf^®^, Chiesi Farmaceutici S.p.A., Parma, Italy). *Poractant alfa* is a natural surfactant, prepared from porcine lungs, containing almost exclusively polar lipids, in particular, phosphatidylcholine (PC, about 70% of the total phospholipid content), and about 1% of specific low molecular weight hydrophobic proteins SP-B and SP-C at a phospholipid concentration of 80 mg/mL.

Surfactant aerosols were generated with the investigational eFlow-Neos vibrating-membrane nebulizers (PARI Pharma, Starnberg, Germany) controlled with the eVent-Neos control unit (PARI Pharma, Starnberg, Germany). The device is able to deliver large doses of undiluted *Poractant alfa* (>1000 mg; or 12.5 mL) at relatively high rates (16 mg of phospholipids/min; or 0.2 mL/min) without clogging the nebulizer membrane [28]. *Poractant alfa* aerosols generated at 37 °C and >95% relative humidity conditions were characterized by a mass median diameter (MMD) of 3.0 ± 0.1 μm, a geometric standard deviation (GSD) of 1.5, and a fine particle fraction (i.e., particles with a diameter below 5 μm) of 93.7 ± 1.1% [13].

### 2.2. High Flow Nasal Cannula (HFNC) Devices 

Three types of commercially available NCs of different sizes were evaluated in the present study: (1) Fisher & Paykel premature (BC2425, red) and neonatal NCs (BC2435, yellow, Fisher & Paykel Healthcare, Auckland, New Zealand), (2) Vapotherm NCs in premature and neonatal sizes (Vapotherm, Exeter, NH, USA), and (3) Westmed NC in infant size (Westmed, Tucson, AZ, USA). 

The flow in the Fisher & Paykel NCs was generated with the Optiflow^TM^ system (Fisher & Paykel Healthcare, Auckland, New Zealand), which incorporates an air–oxygen blender, flow meter, and a heated humidifier, providing gas with a temperature ranging between 33 and 43 °C and 95–100% humidity. A Fabian ventilator (Fabian HFO, Acutronic, Zug, Switzerland) and a Fisher & Paykel humidifier were used to deliver HFNC with the Vapotherm and Westemed NCs. 

### 2.3. Pharyngeal Pressure Measurements

An essential requirement to evaluate the effect of nebulized surfactant delivered during HFNC in surfactant-depleted rabbits was to ensure the compatibility of the neonatal NCs with the rabbit’s upper airway anatomy. Therefore, the fit and insertion stability in the rabbits’ nose and the pharyngeal pressure generated by the different NCs was assessed in surfactant-depleted adult rabbits. The pharyngeal pressure generated during nasal CPAP at a level of 5 cmH_2_O with nasal prongs customized to the rabbits’ nose was used as a reference value. Such nasal prongs have been previously used to deliver nasal CPAP, nasal intermittent positive pressure ventilation (NIPPV), and synchronized NIPPV to surfactant-depleted rabbits [29].

The pharyngeal pressure measurements were carried out in 6- to 7-week-old surfactant-depleted male rabbits (Charles River Laboratories, Calco, Italy). All procedures conducted on animals were approved by the intra-mural Animal Welfare Body and the Italian Ministry of Health (Prot.n° 1300–2015-PR). Animal handling and the surfactant depletion procedure by Broncho-alveolar lavages (BAL) have been described elsewhere [29,30]. Briefly, animals were stabilized in mechanical ventilation (Acutronic Fabian HFO ventilator) with the following settings: fraction of inspired oxygen (FiO_2_) 100%, inspiratory flow = 10 L/min, respiratory rate (RR) = 40 cycles/min, positive-end expiratory pressure = 3 cmH_2_O, tidal volume (*V*_T_) targeted to 7 mL/kg (with a maximum peak inspiratory pressure not higher than 23 cmH_2_O). While intubated, the animals underwent repeated BALs by flushing 20 mL/kg of pre-warmed 0.9% NaCl solution until the partial pressure of arterial oxygen (PaO_2_) dropped below 150 mmHg (with a FiO_2_ of 100%). If the PaO_2_ remained below 150 mmHg in a subsequent blood gas analysis performed 15 min later, the respiratory distress was confirmed, and the animal was included in the study. The rabbits were then extubated and managed with HFNC support with a FiO_2_ of 100% and a gas flow of 8 L/min. 

The pharyngeal pressure generated by the different NCs was determined by placing a customized flow sensor in the pharynx of the rabbits. The pressure signal was amplified, recorded, and analyzed with data acquisition software (PowerLab, ADI Instruments, Colorado Springs, CO, USA). Surfactant-depleted adult rabbits were managed for 5 min with HFNC, and the pharyngeal pressure was annotated. For each NC type, three independent pharyngeal pressure measurements were performed. During pharyngeal pressure measurements, the animal caregivers evaluated the insertion stability and the animal comfort by observational quality criteria. 

### 2.4. Benchmark Surfactant Aerosol Deposition Studies 

**Figure 1:**
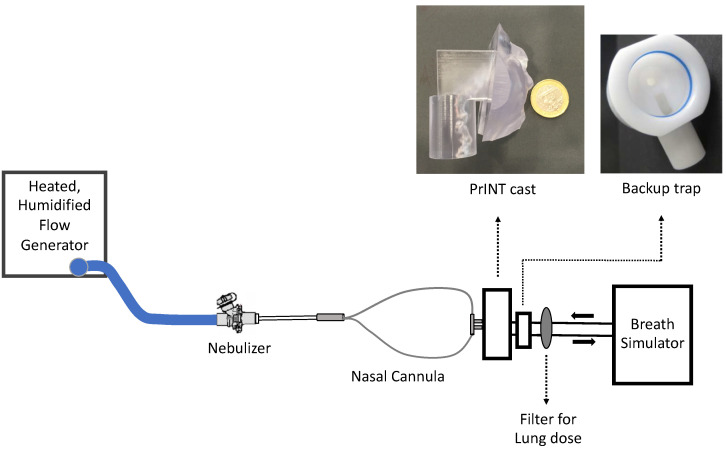
Scheme of the experimental setup. PrINT, Premature Infant Nose Throat-Model.

According to the in vivo compatibility check, the Westmed (infant size) and Fisher & Paykel premature NCs were selected for benchmark studies. The in vitro set-up is depicted in Figure 1 and consisted of a heated, humidified flow-generating device (Optiflow^TM^ or Fabian ventilator, as appropriate), a customized eFlow Neos nebulizer, the corresponding NCs, a 3D-printed cast of the upper airways of a 32 weeks’ gestation premature infant (PrINT model) [31], and a breath simulator programmed with the sinusoidal breathing pattern of a premature infant: *V*_T_ of 8.9 mL, RR of 70 cycles/min and an I:E of 40/60. The nebulizer was placed right before the bifurcation of the NCs. A surfactant-collecting filter (PARI filter PAD, PARI Pharma, Starnberg, Germany) was placed in the distal airway of the PrINT model to estimate the surfactant lung dose. 

The PrINT model resembles the upper airways of a premature infant and is based on the three-dimensional reconstruction of a magnetic resonance image obtained from a baby born at 32 weeks’ gestation, who had a birth weight of 1750 g [31]. Therefore, 350 mg of undiluted *Poractant alfa* (4.375 mL), a dose equivalent to 200 mg/kg for the PrINT neonate, was nebulized in each in vitro experiment. After the full surfactant dose was delivered, the set-up was carefully dissembled, and the surfactant was extracted from each component using a rinsing solvent containing chloroform/methanol 50:50. The combined amount of surfactant deposited in the nebulizer and within the NCs was determined. The surfactant deposited onto the face of the PrINT model was recovered with a tissue, and together with the surfactant extracted from the upper airways of the cast (i.e., before the glottis) represented the total surfactant recovered from the PrINT model. The surfactant collected in the backup trap resembles the fraction of surfactant that impacts the PrINT’s airway and moves forward as a liquid film. Lastly, the surfactant collected in the inhalation filter represents the surfactant aerosol lung dose. A validated high-pressure liquid chromatography (HPLC) method with the appropriate external standard calibration was used for quantifying PC [13], the lead compound of *Poractant alfa*. The experiments with each type of NC were repeated at least three times.

### 2.5. In Vivo Effect of Nebulized Surfactant during HFNC with the Optiflow^TM^ System in Surfactant-Depleted Rabbits

Eighteen surfactant-depleted adult rabbits were allocated to one of the three experimental groups: animals in the “**HFNC** (**Control**)” group (n = 6) were extubated after the confirmation of respiratory distress and managed with HFNC ventilation. Animals in the “**SF Instillation + HFNC**” group (n = 6) received a clinical dose of intratracheal *Poractant alfa* (200 mg/kg) before extubation and were then managed with HFNC. Finally, animals in the “**HFNC + SF** (**400 mg/kg**)” group (n = 6) were extubated and received a nominal dose of 400 mg/kg of nebulized *Poractant alfa* during HFNC ventilation. In all groups, HFNC ventilation was delivered at a flow of 8 L/min via the Optiflow^TM^ system with the Fisher & Paykel red premature NC for 180 min.

Arterial blood gases were withdrawn and analyzed for arterial carbon dioxide partial pressure (PaCO_2_) and PaO_2_ (Radiometer Medical, Copenhagen, Denmark) at baseline (healthy lung status), after the confirmation of respiratory distress (right before extubation), and at regular intervals after shifting to HFNC, at 15 and 30 min after starting the HFNC, and then every 30 min until the end of the experiment. 

At the end of the follow-up period (180 min), all animals were re-intubated and briefly managed with positive pressure ventilation using the same ventilation settings as at baseline. This way, the dynamic compliance (C_dyn_) at baseline (healthy status), after the confirmation of respiratory distress, and after treatment could be compared. C_dyn_ was calculated by dividing lung volume (∆V, in mL) by the changes in pressure (∆P, in cmH_2_O) standardized by the animal’s weight:

C_dyn_ = ∆V/(∆P × Weight)

A pressure/volume curve was performed post mortem by progressively applying 5, 10, 15, 20, 25, and 30 mL of air to the lungs with a precision syringe. The pressure required to reach a 30 mL lung volume was annotated and used to compare the lung mechanics across different groups.

### 2.6. Statistical Analysis

Unless otherwise stated, data are presented as the mean ± standard deviation. Raw data were compared by one-way analysis of variance (ANOVA) or by two-way ANOVA as a function of group and time, followed by Tukey’s post hoc test. Statistical analysis was performed using Prism (GraphPad Software version 7.0, San Diego, CA, USA).

## 3. Results

### 3.1. Pharyngeal Pressure Measurements

**Figure 2:**
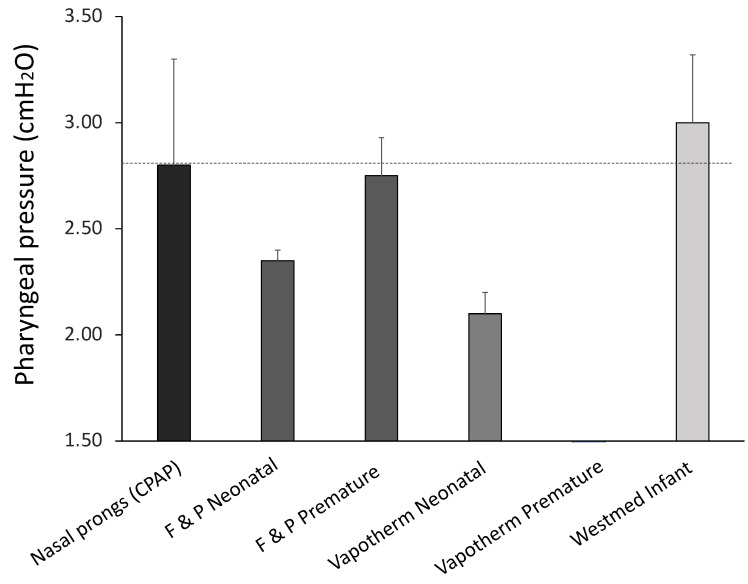
Pharyngeal pressure measurements during humidified high-flow nasal cannula (HFNC) respiratory support at 8 L/min with different types of commercially available nasal cannulas (NCs) in spontaneously breathing adult rabbits with induced respiratory distress. The dashed line indicates the reference pharyngeal pressure achieved at a nasal continuous positive airway pressure (CPAP) level of 5 cmH_2_O. No signal of pharyngeal pressure was detected with the Vapothern premature NCs. Three independent pharyngeal pressure measurements were performed for each NC type.

The outcome of the pharyngeal pressure measurements with different NCs is displayed in Figure 2. The reference pharyngeal pressure during CPAP at 5 cmH_2_O was 2.80 ± 0.5 cmH_2_O. Equivalent pharyngeal pressure values during HFNC ventilation were only achieved by the Fisher & Paykel premature size (2.78 ± 0.18 cmH_2_O) and the Westmed NCs (3.0 ± 0.32 cmH_2_O). The remaining NCs registered pharyngeal pressures below 2.5 cmH_2_O. No signal of pharyngeal pressure was detected with the Vapotherm premature NCs, indicating no compatibility with the rabbit’s upper airway anatomy.

### 3.2. Benchmark Surfactant Aerosol Deposition Studies 

The fraction of surfactant deposited on each part of the in vitro set-up after nebulization with the Westmed and Fisher & Paykel NCs is displayed in Table 1. The lung dose was higher with the Fisher & Paykel premature size NCs compared with the Westmed infant NCs. The highest fraction of surfactant was recovered from the PrINT cast in both cases, although more surfactant was recovered from the PrINT cast with the Westmed NCs. Conversely, more surfactant was deposited within the nebulizer and NCs after nebulization using the Fisher & Paykel NCs. In addition, a higher fraction of surfactant was collected in the backup trap placed between the PrINT and the lung dose filter with the Fisher & Paykel NCs, indicating that more surfactant entered the upper airways of the PrINT cast with this type of NCs. The higher lung dose and the greater fraction of surfactant recovered in the backup trap predicted a higher intra-corporeal aerosol deposition with the Fisher & Paykel NCs, which were, therefore, selected for the subsequent in vivo study. Approximately 25–30% of the surfactant filled in the nebulizer could not be recovered and was most probably released to the ambient air as fugitive aerosols through the air leak occurring between the HFNC interface and the PrINT model.

### 3.3. In Vivo Effect of Nebulized Surfactant during HFNC with the Optiflow^TM^ System in Surfactant-Depleted Rabbits 

There were no significant differences between groups in the body weight or the number of BALs required to induce respiratory distress (Table 2).

**Figure 3:**
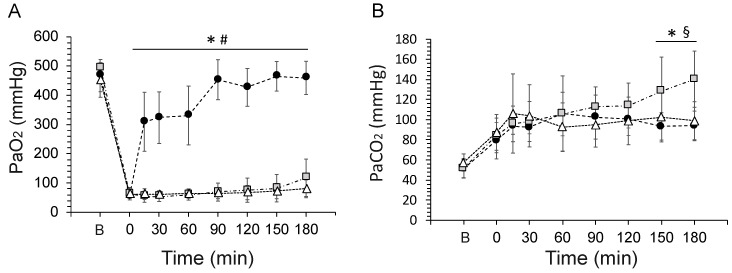
(**A**) Mean partial pressure of arterial oxygen (PaO_2_) and (**B**) the partial pressure of arterial carbon dioxide (PaCO_2_) in surfactant-depleted adult rabbits treated with a humidified high-flow nasal cannula (HFNC) only (grey squares), with 200 mg/kg of intratracheal surfactant (black circles), or with 400 mg/kg of nebulized surfactant in combination with HFNC (white triangles). * Between intratracheal surfactant and HFNC-only groups, *p* < 0.01; ^#^ Between intratracheal surfactant and HFNC + nebulized surfactant (400 mg/kg) groups, *p* < 0.01; ^§^ Between HFNC + nebulized surfactant (400 mg/kg) and HFNC-only groups, *p* < 0.01.

Repeated BALs induced severe respiratory distress, producing a dramatic drop in arterial oxygenation and an increase in the PaCO_2_. Arterial oxygenation rapidly improved in the animals treated with an intratracheal bolus of 200 mg/kg of surfactant and gradually increased over the follow-up period, reaching baseline values at 180 min (Figure 3A). In contrast, the mean PaO_2_ of the animals treated with nebulized surfactant during HFNC did not improve despite receiving a nominal dose of 400 mg/kg. Arterial oxygenation was also low in the HFNC (Control) group, as expected, although a slight increase in the mean PaO_2_ could be observed after 150 min of HFNC ventilation.

All animals developed hypercarbia after surfactant depletion, which was persistent in all the experimental groups (Figure 3B). However, the animals in the HFNC (Control) group registered a steady increase in the PaCO_2_, which reached statistical significance compared to surfactant-treated groups at 150 and 180 min. 

**Figure 4:**
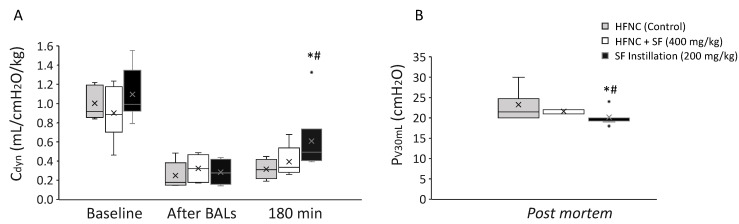
Box plots showing (**A**) dynamic compliance (C_dyn_) in adult rabbits at baseline, after inducing respiratory distress (after BALs) and 180 min after receiving different treatments, and (**B**) pressure registered after applying 30 mL of air (P_V30 mL_) post mortem into the lungs of animals treated with a humidified high-flow nasal cannula (HFNC) only (control, grey boxes), with 200 mg/kg of intratracheal surfactant (SF instillation, black boxes), or with 400 mg/kg of nebulized surfactant in combination with HFNC (white boxes). The boxes display the median (horizontal line) and the first and third quartiles. The *x* within the boxes indicates the mean of each group, and the whiskers display the maximum and minimum values within the dataset. The dots beyond the whiskers represent outlier values. * Between SF instillation and HFNC-only groups, *p* < 0.01; ^#^ Between SF instillation and HFNC + nebulized surfactant (400 mg/kg) groups, *p* < 0.01.

The mean C_dyn_ dropped by over 50% in all groups after surfactant depletion. C_dyn_ improved in the animals treated with an intratracheal surfactant bolus, although the mean C_dyn_ recovery could not reach baseline values. At 180 min, mean Cdyn was significantly higher in the surfactant instillation group compared with the HFNC + SF (400 mg/kg) and the HFNC (control) groups (Figure 4A). Similarly, the mean pressure registered after applying 30 mL of air into the lungs was significantly lower in the group of animals treated with instilled surfactant (20.2 ± 2.1 cmH_2_O) compared with animals receiving nebulized surfactant during HFNC (21.8 ± 3.4 cmH_2_O) and HFNC alone (23.25 ± 4.7 cmH_2_O), indicative of improved lung mechanics after surfactant instillation compared with nebulized surfactant. 

## 4. Discussion

The aim of the present study was to explore the delivery efficiency of surfactant aerosols during HFNC to estimate the potential of this treatment for spontaneously breathing premature infants at high risk of developing RDS. The benchmark study revealed the surfactant lung dose to be relatively low independent of types of NCs (<2%). We further compared, in vivo, the effect of nebulized surfactant delivered during HFNC with the clinical surfactant administration method via intratracheal instillation. The modest lung doses achieved in the benchmark study anticipated the lack of effect seen in the in vivo nebulized surfactant study, where arterial oxygenation and lung mechanics did not improve and were significantly worse than the intratracheal instillation of surfactant.

The aerosol delivery of exogenous surfactant during NRS has been a long-pursued goal of neonatal research. Two recently published randomized, controlled trials have reported the positive effects of nebulized surfactant during NRS. Minocchieri et al. reported a significant reduction in the need for mechanical ventilation in premature infants (GA 29–34 weeks) treated with a nominal dose of 200 mg/kg of nebulized *Poractant alfa* delivered with the eFlow Neos nebulizer [25]. Cummings et al. reported a significant reduction in the intubation rate for surfactant instillation in infants (gestational age 23–41 weeks) receiving 210 mg/kg of nebulized *Calfactant* using a Solarys nebulizer modified with an oral interface, namely a “pacifier adapter”, while NRS was simultaneously delivered with a separate nasal interface [26]. The beneficial effects of nebulized surfactant in both studies were restricted to the more mature premature infants (>31 weeks’ gestation), who have a higher likelihood of less severe RDS. It is worth noting that CPAP was exclusively used in the Minocchieri study during surfactant nebulization and was the predominant NRS type (70% of enrolled infants) in the Cummings et al. study. 

Over the last years, HFNC has gained momentum as a first-line treatment for mild and moderate RDS [3,5]. Therefore, effective aerosol delivery strategies compatible with this NRS modality would be highly desired. However, to the best of our knowledge, aerosol lung deposition in premature infants managed with HFNC remains unknown. Corcoran et al. investigated the aerosol deposition of a radioactive tracer in 18-term infants with congenital cardiac disease (median age 26 days) supported with NCs [32]. The radioactive tracer was delivered using a vibrating-membrane nebulizer (Aerogen Solo) placed right before a corrugated tube connected to the NCs. With a flow of 2 L/min, the estimated lung deposition was 0.46% of the nominal dose, which could be increased to 1.10% by lowering the flow to 0.2 L/min. The authors also reported that pilot studies with a flow of 3 L/min were associated with high levels of nasal deposition. The relatively low lung deposition observed by Corcoran et al. contrasts with the recent single-center, Phase II, uncontrolled trial assessing the safety and efficacy of nebulized surfactant, in which HFNC was one of the permitted NRS modalities for aerosol delivery to premature infants [27]. In this trial, Sood et al. randomized 149 premature infants (24–37 weeks’ gestational age) to either receive 100 or 200 mg/kg of surfactant aerosols (*Beractant*, Survanta, Abbot Laboratories) generated with jet or vibrating-membrane nebulizers during NRS. Compared to retrospective controls, the intervention with nebulized surfactant significantly reduced the need for intubation within 72 h. Notably, the authors reported that all infants receiving surfactant aerosol during HFNC (n = 37) avoided intratracheal intubation in the first 72 h. The authors acknowledged that at their institution, HFNC is used in the early stages of RDS with escalation to nasal CPAP followed by NIPPV with worsening respiratory distress. Moreover, 24 out of 37 infants (64%) managed with HFNC belonged to the 33–37 weeks’ gestational age stratum, who are at a lower risk of developing RDS. Surfactant lung deposition was not reported in the trial, and no benchmark studies of lung dose estimation were referenced. Therefore, the positive outcome of the infants treated with nebulized surfactant delivered with HFNC in the Sood et al. study may be partly explained by the inclusion of premature infants with uncomplicated respiratory distress who would do well just with NRS. 

Several preclinical studies have investigated the aerosol delivery efficiency during HFNC in vitro and in vivo. Sunbul et al. [33] compared the lung dose of albuterol delivered with a vibrating mesh nebulizer (Aerogen Solo), either placed before the humidifier of the Optiflow^TM^ system or proximal to the airway cast of a premature infant (DiBlasi model, based on a 26 weeks’ gestation premature infant [34]). In this study, HFNC ventilation was delivered using infant intermediate NCs (Fisher & Paykel) at a flow of 3 L/min and the breathing pattern of an infant (*V*_T_ 9 mL, RR 50 bpm, and Ti 0.5 s). Placing the nebulizer proximal to the airway cast, an albuterol lung dose of 0.90 ± 0.26% was achieved, which was slightly increased to 1.30 ± 0.17% by placing the nebulizer before the humidifier. Réminiac et al. observed a flow-dependent variation of the aerosol lung deposition after nebulization during HFNC with a vibrating-membrane nebulizer (Aerogen Solo) in both benchmark and in vivo scintigraphy studies conducted in spontaneously breathing macaques (3.2–3.6 kg) [35]. The nebulizer was placed right before the humidifier of the Optiflow^TM^ system. Their benchmark study used a neonatal-size NC placed at the Sophia anatomical infant nose-throat model (SAINT model, based on a 9-month-old infant [36]), which was further connected to a pump that simulated a tidal volume of 25 mL. The amount of radioactive tracer collected in the lung dose filter was significantly reduced by increasing the flow; at a flow rate of 2 L/min, the lung dose was 4.15 ± 1.75% and was, respectively, reduced to 3.29 ± 1.70% and 0.52 ± 0.23% at flows of 4 and 8 L/min. The lung deposition further decreased in spontaneously breathing macaques to, respectively, 0.85 ± 0.57%, 0.49 ± 0.44%, and 0.09 ± 0.04% at 2, 4 and 8 L/min. 

The lung doses achieved in our benchmark study are comparable with the outcomes of the Réminiac et al. study, which also used a flow of 8 L/min. During our benchmark studies, surfactant bubbling could be observed at the outlet of the NCs, regardless of the NC type, and most of the surfactant leaked out of the NCs as a liquid film, depositing in the face of the PrINT model. This observation implies a high impaction of surfactant aerosol particles within the narrow tubing of the NCs, which dramatically reduced the lung dose. Additionally, a surfactant fraction ranging between 25 and 30% was not recovered from the in vitro set-up elements and was most probably released as fugitive aerosols to the ambient air due to the air leaks between the NCs and the nares of the PrINT model. Consequently, the mean lung doses achieved with the Westmed and Fisher & Paykel NCs were dramatically reduced to 0.5% and 1.8% of the nominal dose, respectively. In previous benchmark studies, testing the same drug/device combination in a neonatal nCPAP circuit, we achieved lung doses above 10% [28,37], which correlated well with the lung deposition obtained in scintigraphy studies conducted in neonatal piglets managed with nCPAP (mean surfactant lung deposition of 15.9 ± 11.9%) [17]. These differences between nCPAP and HFNC may be partly explained by the position of the nebulizer within the setup, which was placed as close to the patient as possible during nCPAP experiments, between the nasal prongs and the connection of the ventilator circuit, to avoid aerosol dilution by bias flow. Conversely, in the present study, the nebulizer was placed right before the NCs, which promoted the impaction of aerosol particles within the narrow NC tubing, thereby reducing the lung dose. Moreover, it is worth mentioning that the bias flow was lower during CPAP experiments (5 L/min vs. 8 L/min in the present study) and that air leaks at the patient interface were minimized during nCPAP experiments by sealing the CPAP interface to the nares of the PRiNT cast with silicon, whereas in the present study, air leaks around the nasal interface were allowed. 

The delivery of a nominal dose of surfactant of 400 mg/kg to surfactant-depleted rabbits during HFNC did not show any beneficial effects in terms of arterial oxygenation or lung mechanics, in line with untreated controls. The 400 mg/kg dose was chosen based on the observation by Lewis et al., who described a pulmonary improvement in preterm lambs with as low as 2 mg/kg of surfactant depositing in the lungs after nebulization [38], an intrapulmonary surfactant dose strikingly lower compared with the currently approved surfactant instillation protocols (100–200 mg/kg). The different distribution patterns may explain a higher efficacy of lower intrapulmonary surfactant doses after nebulization; instilled surfactant tends to follow gravity, whereas surfactant aerosols follow ventilation. Therefore, with an expected lung deposition of 0.5–1.8%, one could estimate an intrapulmonary surfactant dose ranging between 2 and 8 mg/kg with a 400 mg/kg nominal dose, in the range of the intrapulmonary surfactant dose reported by Lewis et al. to elicit a lung response. However, as previously reported by Réminiac et al. [35], the lung dose determined in vitro may have overestimated the actual surfactant deposition in spontaneously breathing rabbits. The outcomes of the present study contrast with previous studies in surfactant-depleted adult rabbits and neonatal piglets managed with nCPAP in which nominal doses of nebulized surfactant as low as 100 mg/kg achieved a slight but significant improvement of PaO_2_ [13], and doses of 200 and 400 mg/kg were associated with a pulmonary response equivalent to the intratracheal instillation of a clinical dose of surfactant (200 mg/kg) in terms of gas exchange and lung mechanics [13,15]. Thus, the in vivo results obtained in the present study indicate that nCPAP outperforms HFNC as an NRS type for surfactant administration with the current drug/device combination. 

This study has some limitations. We used commercially available NCs designed for neonates, which were applied to adult rabbits with different upper airway anatomy. Therefore, a flow of 8 L/min was required to achieve a pharyngeal pressure equivalent to that recorded during nCPAP at 5 cmH_2_O. Lower flows could likely achieve slightly higher lung doses. Air flows ranging between 2 and 8 L/min have been applied to neonates managed with HFNC, reaching nasopharyngeal end-expiratory pressures of 6.1 ± 2.1 cmH_2_O at 8 L/min [6]. The pharyngeal pressures measured in our rabbit model at 8 L/min during HFNC were lower (<3 cmH_2_O) than in human neonates and may partially explain the high PaCO_2_ values in all groups, including the group of animals treated with instilled surfactant. The limited sample size and the use of 100% FiO_2_, which is associated with oxidative stress in preterm neonates [39], are also important limitations of the study.

## 5. Conclusions

The present study investigated the lung dose and the pulmonary effects in vivo of nebulized surfactant during HFNC. The in vitro study revealed relatively low surfactant lung doses, irrespective of the NC type used (<2% of the nominal dose). No signs of pulmonary efficacy were observed after the nebulization of a 400 mg/kg surfactant dose delivered to surfactant-depleted rabbits with respiratory distress. Conversely, the intratracheal instillation of a clinical dose of surfactant (200 mg/kg) significantly improved arterial oxygenation and lung mechanics. The lack of in vivo effect may be explained by the low surfactant deposition in the animals’ lungs, which primarily occurred during nebulization via HFNC due to the impaction of a large fraction of the aerosol in the NCs, and also by the release of a significant amount of the surfactant aerosol dose to the ambient air through the air leak occurring at the HFNC interface. The results of the present study discourage the use of HFNC as a non-invasive respiratory support modality for the delivery of nebulized surfactant under the described configuration

## Figures and Tables

**Table 1:** Surfactant deposition during humidified high-flow nasal cannula (HFNC) at 8 L/min.

Surfactant Deposition (%)	Westmed (Infant Size)	Fisher & Paykel (Premature Size)
PrINT cast	66.9 ± 5.9	46.2 ± 7.9
Nebulizer + nasal cannula	5.5 ± 0.3	18.8 ± 4.1
Backup trap	0.6 ± 0.1	4.2 ± 4.1
Lung dose	0.5 ± 0.45	1.8 ± 1.9
Surfactant recovered vs. filled	73.5 ± 6.0	71.0 ± 14.8

PrINT, Premature Infant Nose Throat-Model.

**Table 2:** Mean weight and number of bronchoalveolar lavages performed in the experimental groups.

	Body Weight	Number of BALs
HFNC (Control)	1.80 ± 0.06	11.33 ± 1.56
SF Instillation + HFNC	1.88 ± 0.12	11.16 ± 1.56
HFNC + neb SF (400 mg/kg)	2.05 ± 0.12	9 ± 1.23

Mean ± standard error of the mean. HFNC, humidified high-flow nasal cannula; SF, surfactant; neb, nebulized; BALs, bronchoalveolar lavage.
